# Fatigue Monitoring in Running Using Flexible Textile Wearable Sensors

**DOI:** 10.3390/s20195573

**Published:** 2020-09-29

**Authors:** Mohsen Gholami, Christopher Napier, Astrid García Patiño, Tyler J. Cuthbert, Carlo Menon

**Affiliations:** 1 Menrva Research Group, Schools of Mechatronic Systems & Engineering Science, Simon Fraser University, Metro Vancouver, BC V5A 1S6, Canada; mgholami@sfu.ca (M.G.); cnapier@sfu.ca (C.N.); agarciap@sfu.ca (A.G.P.); cuthbert@sfu.ca (T.J.C.); 2 Department of Physical Therapy, Faculty of Medicine, University of British Columbia, Vancouver, BC V5T 1Z3, Canada

**Keywords:** fatigue running, kinematics, soft sensors, random forest

## Abstract

Fatigue is a multifunctional and complex phenomenon that affects how individuals perform an activity. Fatigue during running causes changes in normal gait parameters and increases the risk of injury. To address this problem, wearable sensors have been proposed as an unobtrusive and portable system to measure changes in human movement as a result of fatigue. Recently, a category of wearable devices that has gained attention is flexible textile strain sensors because of their ability to be woven into garments to measure kinematics. This study uses flexible textile strain sensors to continuously monitor the kinematics during running and uses a machine learning approach to estimate the level of fatigue during running. Five female participants used the sensor-instrumented garment while running to a state of fatigue. In addition to the kinematic data from the flexible textile strain sensors, the perceived level of exertion was monitored for each participant as an indication of their actual fatigue level. A stacked random forest machine learning model was used to estimate the perceived exertion levels from the kinematic data. The machine learning algorithm obtained a root mean squared value of 0.06 and a coefficient of determination of 0.96 in participant-specific scenarios. This study highlights the potential of flexible textile strain sensors to objectively estimate the level of fatigue during running by detecting slight perturbations in lower extremity kinematics. Future iterations of this technology may lead to real-time biofeedback applications that could reduce the risk of running-related overuse injuries.

## 1. Introduction

Running is one of the most popular and healthy activities [1,2] worldwide but carries a high risk of injury [3]. Long-distance running is, by nature, a prolonged and repetitive activity, which can induce fatigue. Fatigue accumulates as runners increase distance or intensity and is defined as an exercise-induced reduction in the ability to generate muscle force or power that is caused by changes in the neural drive or exhaustion of muscle contractile function [4]. 

Fatigue is commonly measured either by direct physiological means (e.g., heart rate, blood lactate concentration, etc.) or by subjective rating of exertion. Perceived rating of exertion is a subjective indication of fatigue that integrates information from the peripheral muscles, the central neural system, and the cardiovascular system [5]. The Borg Rating of Perceived Exertion (RPE) scale [6] is a popular and practical tool that has been widely used in running research [7,8,9]. RPE scores are strongly correlated with blood lactate concentration and can be used both in the laboratory and in the field [10,11,12]. A recent systematic review reported that RPE is a more sensitive measure of acute stress (fatigue) than objective measures, such as blood lactate concentration and heart rate [13], as it encompasses both the psychological and physiological components of fatigue [14]. Rating of Perceived Exertion has been increasingly used in running injury research, as well as in clinical contexts, because of its important contribution to the overall calculation of training load—and, therefore, injury risk—for an individual [15,16].

Fatigue can affect both performance and injury risk, manifested by changes in lower extremity biomechanics—particularly joint kinematics. Changes in kinematics and the shift in work from distal to proximal joints [17] is believed to reduce running economy during runs to exhaustion [18,19]. Running in a fatigued state also exposes the body to greater mechanical forces, which may increase risk of injury [20,21,22]. The greater loads experienced by the lower extremities when fatigued are due to changes in kinematics at initial contact and during the early loading phase of running [23,24,25,26].

Kinematic [20,26,27], kinetic [28,29], and electromyographic (EMG) [30,31,32] changes may occur in response to fatigue. Traditionally, these changes have been measured by optical motion capture systems, force plate or accelerometer analysis, and surface EMG (sEMG), respectively. While kinetics and EMG can be measured remotely (i.e., outside of a lab environment), the remote measurement of kinematics is more challenging. The concept that increasing fatigue during a prolonged run is correlated with a change in kinematics is not new [20,26,27]. Assuming that the kinematic changes are as predictable (and progressive) as the level of fatigue, the fatigue level of a runner could be estimated by continuously monitoring their kinematics using a wearable device. From a machine learning perspective, the amount of information gathered from lower extremity kinematics may yield a better estimation of fatigue than monitoring kinetics or EMG activity. As kinematic changes associated with fatigue may decrease performance and increase injury risk [33], the ability to monitor changes in kinematics over the course of a run, especially outside of a lab or clinic setting, has substantial implications on both performance and injury risk. 

Two main methods have been used to measure changes in kinematics to detect fatigue: (1) optical motion capture and (2) wearable devices. Wearable sensors have the advantage of unobtrusive measurement in ecologically valid environments during daily life. Inertial measurement units (IMUs) are the primary wearable device that have been used to detect the state of fatigue or to quantitatively measure fatigue level. A machine learning approach has been used to estimate the level of fatigue based on information from wearable sensors, including Strohrmann et al.’s [34] investigation into the correlation of features extracted from IMUs with the level of fatigue during a prolonged run. There was a strong correlation between IMU signal changes from 12 IMUs mounted on the lower and upper body and the perceived level of exertion of runners. Other studies have focused on the binary detection of the state of fatigue and non-fatigue based on measurements by IMUs [35,36]. Tibia-mounted IMUs have obtained a high classification accuracy of fatigue during running and occupational tasks [36]. Karg et al. [37] used a hidden Markov model as a regression model to predict fatigue level during the performance of a squat exercise. This study monitored the kinematics of the lower and upper body using an optical motion capture system and obtained a high accuracy level. 

To our knowledge, the use of flexible textile (soft) strain sensors for continuous monitoring of fatigue has not yet been studied. Flexible textile strain sensors are a category of wearable sensors that have recently been used for human gait analysis [38,39,40], trunk motion monitoring [41,42], and hand gesture recognition [43]. These sensors work by measuring changes in the resistance or capacitance when they are elongated [44]. The advantages of flexible textile strain sensors are their easy integration into clothing and convenient use compared to rigid IMUs. In a previous study, we optimized a wearable flexible textile strain sensor system for three dimensional lower extremity joint angle measurement and subsequently validated this system for the measurement of lower extremity running kinematics [38]. In this study, we extend our previous research to use flexible textile strain sensors for the estimation of fatigue level during running. Our aim was to estimate the level of fatigue (measured by Borg’s RPE scale) during the course of a prolonged run based on the lower extremity kinematic information provided by the flexible textile strain sensors. We hypothesized that by using information from the flexible textile strain sensors and using a machine learning approach, we would be able to accurately estimate the level of fatigue at a given moment for an individual during a prolonged run.

## 2. Materials and Methods

### 2.1. Experiment Setup

A resistance-based flexible textile strain sensor was employed in this study. The strain sensors were made of spandex multifilament yarn coated with a carbon black thermoplastic elastomer composite [41]. The sensors were conditioned with sinusoidal strain of 40% at 10% per second before use. The sensor showed highly linear performance and no hysteresis in the working range of up to 30% strain. The sensor was then coated with an insulating sheath to prevent shortening of the circuit if participants sweat during the prolonged run. The sensor characteristics are summarized in Table 1.

The resistance-based strain sensor’s fundamental work is expressed in Equation (1), where the resistance value is affected by the increase in the sensor’s length:

(1) $$\Delta R=\frac{\Delta L}{L_{0}}$$

**Figure 1:**
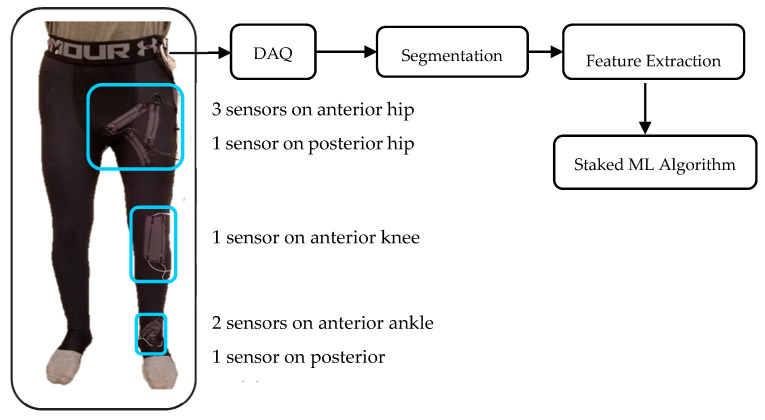
Strain sensor positions on a pair of running tights and the data processing schematic. DAQ, data acquisition device; ML, machine learning.

The sensor placement was optimized in a previous study [38] and four positions were selected by genetic algorithm for a multi-axis hip joint. The positions of the sensors on the knee and ankle were selected according to the joint axes. The wearable system was validated for lower extremity joint angle measurement during running and showed an error of less than 2.5° for multi-axis lower body kinematic monitoring during running [38]. The prototype had a total of nine sensors: four sensors at the hip, two sensors at the knee, and three sensors at the ankle. However, one of the knee sensors was excluded from data processing because of high noise. The circuit used for data recording was a voltage divider circuit with a resistance of 40 kΩ. Participants were asked to run on a split-belt treadmill (Bertec Corporation, Columbus, OH, USA) while the strain sensor signals were recorded at a frequency of 100 Hz by a data acquisition device. The prototype and the schematic of data processing are outlined in Figure 1. The raw signals of the sensor did not drift over the course of 45 min of running. Similarly, the participant’s sweat did not affect the performance of the sensor. 

### 2.2. Data Collection

Five healthy, pain-free female recreational runners participated in this study. The selection of female participants was due to known differences in kinematics between male and female runners [45,46]. To obtain a homogenous sample, participants were selected based on running experience and recent performance in a 10-kilometer race. Participants were also screened to ensure adequate fit of the prototype garment. Onset of the COVID-19 pandemic and the restrictions on in-person research limited data collection to a sample size of five.

**Figure 2:**
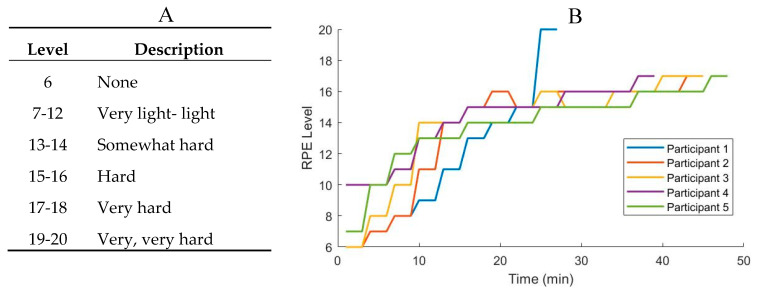
(**A**) Description of levels of Borg Rating of Perceived Exertion (RPE) scale and (**B**) changes in the Borg RPE of each participant during the test.

Participants ran in standardized running shoes (New Balance 880v10, Boston, MA, USA) to ensure that biomechanical changes were not due to differences in shoe characteristics. The use of the Borg RPE scale was explained to the participants prior to the experiment. For consistency, participants were asked to report “the conscious sensation of how hard you are driving your working limbs and how strenuous the exercise feels at this point in time.” After putting on the instrumented running tights, participants were given three minutes to warm up and become familiar with the treadmill, running at a steady speed of 8 km/h. The treadmill was then stopped, and participants were asked to rate their current level of exertion on the Borg RPE scale before commencing the test. Data collection began with participants running at 8 km/h. Participants were asked to report their RPE score every three minutes. If the RPE score was lower than 13 (“somewhat hard”), the speed was increased by 1 km/h. Once the participant reached an RPE score of 13, the speed was kept constant and they were asked to continue to report their RPE score every three minutes until they reported a level of 17 (“very hard”). At this point, one more minute of data was recorded before terminating the test. Figure 2B shows the individual changes in the participants’ RPE scores during the test. 

### 2.3. Data Segmentation

**Figure 3:**
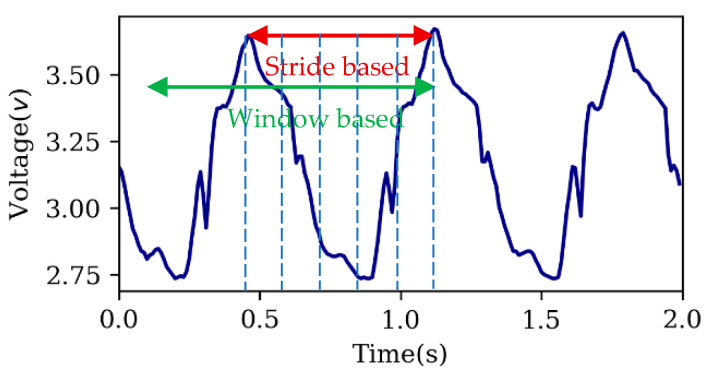
The window-based and stride-based approached for sensor data segmentation.

Two different methods were used to segment the strain sensor signals: (1) based on strides and (2) based on a moving window over the data. In method (1), the peak values of the first sensor were detected and used to determine strides. In method (2), the peak values of the first signal were detected and a window of time prior to this data point was selected. Window lengths of 0.5, 1, 1.5, and 2 seconds were examined. The window length of 1 second obtained the best results (Figure 3).

### 2.4. Feature Extraction

A set of statistical and temporal features were extracted according to the biomechanical changes of the lower extremity associated with fatigue. The features were: mean; minimum; maximum; range of motion; stride length—in addition, variation of mean; minimum; maximum; range of motion during the last 10 strides/windows. Moreover, the strides/windows were segmented to five sub-segments and the mean, minimum, and maximum values of each sub-segment were extracted. The same features were extracted from the first derivative of the strain sensor’s signals. 

### 2.5. Machine Learning Algorithm

Random forest is an ensemble of decision trees that has shown promising results when compared with conventional machine learning algorithms including support vector machine and neural networks in regression and classification applications for strain sensor’s data analysis [39,47]. Random forest models are robust to outliers, nonlinear and unbalanced data, and produce low bias and moderate variance [48,49]. We have previously used random forest to accurately estimate joint angles using strain sensors [39]. We therefore chose random forest as our method for data analysis. Deep learning models were not employed for this study since they typically require large data sets for optimal performance and the limited data used in this study would not be favorable for those models. However, we have previously shown the potential of deep convolutional neural networks to predict joint kinematics if there are a substantial amount of data that allow the models to work efficiently [38]. A two-step regression model (called the first and second model) was used for estimation with each random forest regressor having 100 ensemble trees. The first model was trained using the extracted features from eight strain sensors and the second model was trained based on the predicted values of the first model. The first model was intended to make a rough estimation of exertion level while the second model could perfectly match the initial prediction to the reference value. The second model could particularly help in cases where the first model interpolated an RPE value between the scores that each participant reported during the test. Considering that the RPE score of a participant is unlikely to vary over two successive strides, two subsequent voting windows with a length of 10 samples were moved over the predicted fatigue level and the median predicted value under the windows was considered as the fatigue level. The value selected by the window was then rounded to result in an integer RPE score.

## 3. Results

The extracted features were fed into a random forest algorithm and the performance of the model in a five-fold cross-validation method was investigated. Two different metrics were used to compare the performance of the machine learning regressor: R-squared (R^2^) value and root mean squared error (RMSE) values. Table 2 shows the performance of the algorithm for five participants based on the window-based segmentation method. An average R^2^ value of 0.96 and an RMSE of 0.06 were obtained with this method. 

**Figure 4:**
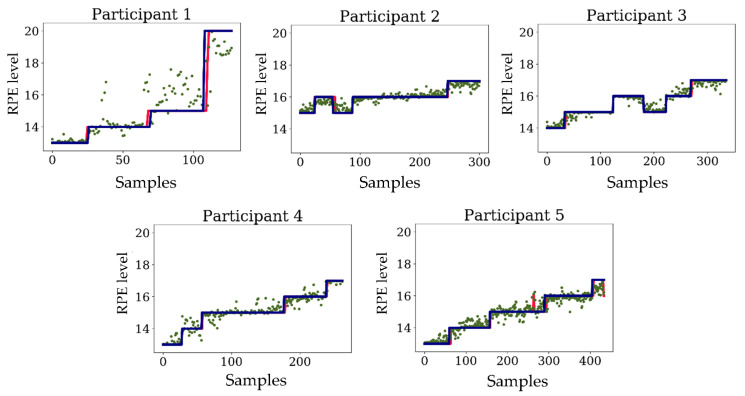
The output of the first level regressor and final prediction of RPE level.

Figure 4 shows the predicted and reference first and second model RPE scores during the prolonged run for all participants. The estimated values calculated by the first model are scattered around the reference value. However, the final prediction followed the reference RPE score. The RPE score did not follow a progressive increase during the prolonged run for participants 2 and 3, yet the predicted values follow the changes in the participants’ RPE scores. 

### Sensor Location Importance

**Figure 5:**
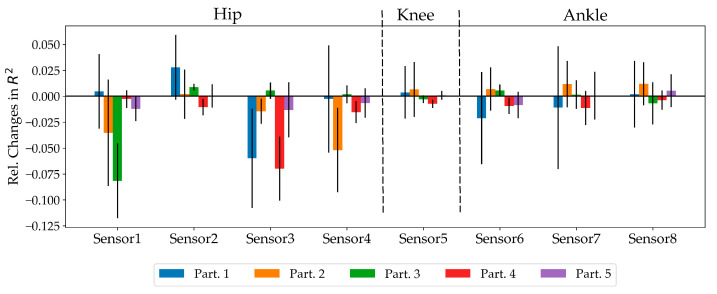
Relative changes in the coefficient of determination by excluding all features from a sensor for each participant.

The importance of the different sensor locations was evaluated by relative changes in R^2^ while excluding all the features that were extracted from a specific sensor (Figure 5). Greater negative values represent increased importance of a sensor. Comparing the importance of each sensor across all participants indicated that the changes in kinematic parameters between different participants were the main difference. However, on average, the sensors placed on the hip reported a greater relative reduction in R^2^ value.

To better understand how changes in kinematics at different joints contribute to the overall estimation of fatigue, features from the sensors on the hip, knee, and ankle were individually fed into a random forest machine learning algorithm. Table 3 shows the results of this analysis using the same two-step regressor. The results from using only the hip sensors are close to the overall accuracy, with only a 0.01 reduction in the coefficient of determination (R^2^) and 0.02 increase in RMSE. However, the results when only relying on the knee or ankle sensors were substantially worse. The ankle sensors contributed less than other joints and could only obtain an R^2^ of 0.10. 

## 4. Discussion and Conclusions 

In this study we investigated the ability of flexible textile wearable sensors to monitor and predict the rating of perceived exertion over the course of a prolonged run. Flexible textile sensors have been previously studied for human motion monitoring [38,41,42]. The wearable system used in this study has been previously validated for lower body kinematic monitoring in running, and sensor positions on the hip were optimized [38]. In contrast to other previous studies investigating fatigue prediction [35,36], we have attempted to predict the rating of perceived exertion discretely and continuously instead of merely detecting a binary state of fatigue or no fatigue. Using a random forest machine learning algorithm, we were able to successfully predict the RPE score of each participant throughout a prolonged run ranging from 30 to 50 minutes.

A machine learning approach to estimate the level of fatigue during running has been demonstrated previously by Khan et al. using sEMG and blood lactate analysis [50]. However, this study only segmented fatigue into three classes (aerobic, anaerobic, and recovery phases based on blood lactate levels) whereas we were able to estimate fatigue accurately over a range of RPE from 13 to 20. The advantage of our method is that fatigue can be more discretely predicted, which allows for graded monitoring. This method has the potential to be used in biofeedback to report the level of fatigue in real-time, lending itself to performance and injury risk applications. Our method using strain sensors showed a strong correlation between lower extremity kinematics and RPE level for all participants (R^2^ > 0.95). The importance of the second level regression is in mapping the rough estimation of the first level regressor (Figure 4) to the RPE level. The first level regressor interpolates between the existing RPE levels and therefore its results are scattered around true RPE levels (e.g., Figure 4, Participant 1). However, the second level regressor learns how to map the interpolated values to the exact RPE levels. 

The contribution of different sensors to the final prediction values was highly participant-specific, indicating the individualized nature of fatigue. When comparing the contribution of different joint kinematics to the overall estimation of RPE, we found that we were able to obtain an R^2^ of 0.95 using only the hip sensors which was one percent less than when using all sensors. Conversely, we observed a significant reduction in the coefficient of determination while using only knee or ankle sensors. Previous studies have reported large increases in ankle frontal plane motion after an exhaustive run [20,27], but as our sensor configuration was primarily oriented to detect changes in ankle sagittal plane motion, the ability of the ankle sensor to detect these large changes was limited. Furthermore, peak knee flexion decreases during stance but increases during swing, resulting in minimal changes to overall sagittal plane knee excursion through the gait cycle [26]. In contrast, while peak hip flexion increases (~7%) during an exhaustive run, hip extension decreases by a much larger degree (~28%), [26] which may explain why the hip sensors in our study accounted for the majority of the information regarding level of fatigue. Given the importance of the hip sensors to the overall detection of fatigue, it is possible that a garment using only hip sensors (i.e., tight-fitting shorts) would maintain the strong level of fatigue prediction, while potentially being more attractive to the user (e.g., in warmer weather).

Flexible textile strain sensors provide a convenient approach to measuring biomechanical changes resulting from fatigue. Gold standard methods, including force plate analysis or optical motion capture systems, are challenging for running applications because of the restriction of data collection to lab settings [51]. Other methods that allow for collection outside of the lab, including sEMG, can be plagued with poor signal quality from the difficulty of maintaining appropriate contact with the skin during prolonged vigorous movements. Monitoring kinematics to estimate fatigue has been extended to other activities outside of running. Karg et al. [37] used optical motion capture to monitor kinematics and proposed using a hidden Markov-based approach to continuously monitor fatigue while performing a squat. The authors were able to obtain an RMSE value of 0.4 and R^2^ of 0.90 with this method. Our results are comparable with their findings but have the advantage of being able to monitor participants “in field”. Strohrmann et al. [34] showed that there is an average correlation coefficient of 0.90 between features extracted from IMUs mounted on the lower and upper body. Our findings indicate that an array of four strain sensors mounted only on the hip has the potential to achieve even better results (R^2^ = 0.95). These sensors can be sewn into running garments (such as tights or shorts) that are more likely to be worn by a runner. One major limitation of estimating fatigue level is the variability of kinematic changes between individuals [17,26]. For this reason, most of the previous studies have proposed intra-participant models [37,50,52]. However, using a motor learning approach may overcome this limitation and we aim to investigate this in future studies with larger and more diverse samples. Another perceived limitation of this study is the use of a self-reported measure of fatigue (Borg RPE scale) as opposed to a physiological measure, such as blood lactate level. However, RPE has been strongly correlated with blood lactate level and is less invasive [10,11]. The Borg RPE scale has been used frequently in previous running studies as a self-reported measure of fatigue [7,8,9,12,16]. Variations on this scale have also been proposed to be better estimates of training load than other objective measures such as blood lactate concentration and heart rate [12,13,15]. This is thought to be due to the subjective (emotional) components of fatigue which are believed to be intricately involved with the peripheral components (e.g., glycogen depletion) in the development of the sensation of fatigue [53,54]. We also defined “perceived effort” to the participants prior to the prolonged run and provided descriptive anchors for the number ratings to minimize the subjectivity of RPE [55]. 

## 5. Conclusions and Future Works

This was the first study to propose and evaluate a method using resistance-based flexible textile strain sensors to monitor and predict fatigue in running. We successfully predicted the rating of perceived exertion in all participants during a prolonged run using a pair of previously validated instrumented running tights and a random forest machine learning algorithm. These findings have the potential to offer an objective measurement of fatigue that is non-invasive and based on kinematic changes during a prolonged run. Our results may lead to the development of real-time biofeedback applications that have the potential to improve performance and prevent injury among runners. Future studies should investigate the potential of using flexible textile wearable sensors for training general models and improving inter-participant results. Researchers should investigate the potential of monitoring bilateral lower extremity kinematics and heart rate using strain sensors to find a more generalizable fatigue estimation model.

## Figures and Tables

**Table 1:** Characteristics of the strain sensor.

Characteristics	Condition
Linearity	Highly linear < 30% strain
Hysteresis	Zero for < 30% strain
Frequency response	up to 10 Hz
Gauge factor	5
Signal drift	No drift up to 4 h

**Table 2:** Coefficient of determination (R^2^) and root mean squared error (RMSE) of predicted RPE level with standard deviations.

	Participant 1	Participant 2	Participant 3	Participant 4	Participant 5	Average
**R^2^**	0.97 (0.03)	0.93 (0.01)	0.98 (0.01)	0.98 (0.01)	0.95 (0.01)	0.96 (0.02)
**RMSE**	0.18 (0.22)	0.02 (0.00)	0.02 (0.01)	0.02 (0.01)	0.06 (0.02)	0.06 (0.06)

**Table 3:** The coefficient of determination and root mean squared value of predicted values while using a single joint sensor with standard deviations.

	Hip Sensors	Knee Sensors	Ankle Sensors
**R^2^**	0.95 (0.02)	0.10 (0.42)	0.29 (0.81)
**RMSE**	0.08 (0.08)	0.44 (0.50)	0.34 (0.20)
